# In-line filtration minimizes organ dysfunction: New aspects from a prospective, randomized, controlled trial

**DOI:** 10.1186/1471-2431-13-21

**Published:** 2013-02-06

**Authors:** Martin Boehne, Thomas Jack, Harald Köditz, Kathrin Seidemann, Florian Schmidt, Michaela Abura, Harald Bertram, Michael Sasse

**Affiliations:** 1 Department of Pediatric Cardiology and Intensive Care Medicine, Hannover Medical School, Carl-Neuberg-Strasse 1, 30625, Hannover, Germany

**Keywords:** In-line filtration, Intensive care, Particle, Inflammation, Children, Organ dysfunction

## Abstract

**Background:**

Infused particles induce thrombogenesis, impair microcirculation and modulate immune response. We have previously shown in critically ill children, that particle-retentive in-line filtration reduced the overall complication rate of severe events, length of stay and duration of mechanical ventilation. We now evaluated the influence of in-line filtration on different organ function and thereby elucidated the potential underlying pathophysiological effects of particle infusion.

**Methods:**

In this single-centre, prospective, randomized controlled trial 807 critically ill children were assigned to either control (n = 406) or filter group (n = 401), the latter receiving in-line filtration for complete infusion therapy. Both groups were compared regarding the differences of incidence rates and its 95% confidence interval (CI) of different organ dysfunction as defined by the International Pediatric Sepsis Consensus Conference 2005.

**Results:**

The incidence rates of respiratory (−5.06%; 95% CI, −9.52 to −0.59%), renal (−3.87%; 95% CI, −7.58 to −0.15%) and hematologic (−3.89%; 95% CI, −7.26 to −0.51%) dysfunction were decreased in the filter group. No difference was demonstrated for the occurrence rates of cardiovascular, hepatic, or neurologic dysfunction between both groups.

**Conclusions:**

In-line filtration has beneficial effects on the preservation of hematologic, renal and respiratory function in critically ill patients. The presented clinical data further support our hypothesis regarding potential harmful effects of particles. In critically ill patients infused particles may lead to further deterioration of the microcirculation, induce a systemic hypercoagulability and inflammation with consecutive negative effects on organ function.

**Trial registration:**

ClinicalTrials.gov number; NCT00209768

## Background

Infusion of particles is a side effect of intravenous therapy [1-3]. Particulate contamination is inherent to the applied drug formulation. It arises from drug incompatibility reactions, incomplete reconstitution of drugs, or components of the infusion systems [4]. On an intensive care unit, up to one million particles may be infused per day [3] depending on the complexity and quantity of the infused solutions [5]. Different mechanisms of particle damage to various organs have been proven either by experimental or clinical studies. These include mechanical blockage of small–diameter arterioles and capillaries [6], activation of platelets, neutrophiles and/or endothelial cells [1] with a subsequent generation of occlusive micro-thrombi [7], and granuloma formation via foreign-body giant cell production [7,8]. Particles modulate the immune response [5] and lead to a deterioration of the microcirculation by loss of functional capillary density [6].

In-line filters have been shown to almost completely prevent particles from being infused [9,10] and are able to retain air, endotoxins and micro-organisms such as bacteria and fungal spores [11]. First reports on the clinical relevance of infused particles have been published in 1960 [12,13]. Ever since, their pathogenetic effect on critically ill patients as well as the clinical value of particle retentive in-line filtration have been a matter of ongoing discussion [8]. Recently, several clinical studies revealed new insights in in-line filtration and its benefit for critically ill patients. In a single center trial involving 88 neonates van Lingen et al. found a significant reduction in the incidence of typical neonatal complications by the use of 0.2 μm in-line filters [14]. Consistent with these findings, we demonstrated a significant benefit of in-line filtration in a prospective, randomized, controlled trial including more than 800 critically ill children. In-line filtration was effective in reducing the overall complication rate of severe events such as systemic inflammatory response syndrome (SIRS), sepsis, thrombosis, and organ failure. Length of stay (LOS) and duration of mechanical ventilation on a pediatric intensive care unit (PICU) were also shortened when in-line filtration was used [15].

In the current study we further analyzed the influence of in-line filtration on organ dysfunction which had not been investigated in the first analysis (ClinicalTrials.gov number; NCT00209768). The organ dysfunction criteria were developed by the International Pediatric Sepsis Consensus Conference 2005 [16] to asses changes or deterioration of organ function. Additionally to the primary and secondary endpoints, in cases where criteria for organ dysfunctions were met, this has been prospectively documented for each patient.

The purpose of this study was to further elucidate the clinical impact of in-line filtration on the different organ systems and thereby give further insights into potential pathophysiological effects of particle infusion.

## Methods

### Study design

This single-centre, prospective, randomized, controlled trial was conducted in an interdisciplinary PICU of a university hospital. The ethical committee of Hannover Medical School approved the study protocol. Sponsorship was provided by a research grant of Hannover Medical School. Additional funding was supplied by an unrestricted grant from Pall, Dreieich, Germany and B. Braun Melsungen, Germany. Both companies supplied in-line filters.

### Patient enrolment and randomization

In total, 2542 patients below the age of 18 years admitted to PICU were initially assessed for eligibility. Expected death within 48 hours of admission, recruitment for other trials and absence of any intravenous therapy led to an exclusion from the study (n = 88). Written informed consent was obtained for each child from their legal guardians on admission. In 38 subjects an informed consent was not available due to foreign language or ethical reasons. In 36 patients the guardians refused to participate in the study and in 1233 cases the guardians were not available at the time of admission to obtain the informed consent for participating in the study. Finally, 1147 children were randomized based on a computer generated simple unrestricted randomization list. These patients were randomly allocated to either control (n = 565) or filter (n = 582) group. Length of stay shorter than 6 hours on PICU was predefined as exclusion criterion. 313 children (n = 150 of the control, n = 163 of the filter group) were discharged from PICU within 6 hours and for that reason excluded. 8 children allocated to the filter group and one patient allocated to the control group did not receive the intended intervention and in 4 patients of the filter group the intervention was discontinued. 14 patients (n = 8 of the control, n = 6 of the filter group) were excluded during final validation due to missing of relevant clinical data in their charts. In total, 807 patients (406 control, 401 filter group) were included in the final analysis.

### In-line filtration

Infusion regimen was standardized for all patients in both groups before launching the study. All solutions and medications were prepared according to manufacturer’s instructions. Certain drugs (special antibiotics, antiviral drugs, antimycotics, chemotherapy) and parenteral nutrition were supplied by a centralized intravenous additive service to guarantee chemical stability and aseptic standards. Before the study a computer based program for the analysis and prevention of possible drug incompatibilities (KiK 3.0; oData, Rastede, Germany) was used to optimize the infusion regimen [4]. This optimized infusion regimen was used for filter and control group. The filter group received in-line filtration throughout infusion therapy. In this group all medications and fluids except blood, plasma proteins or fresh frozen plasma were administered via an in-line filter. 0.2 μm pore size positively charged filters (ELD96LLCE/ NOE96E; Pall, Dreieich, Germany) were used for aqueous solutions and 1.2 μm pore size filters (Intrapur Lipid/ Intrapur Neonat Lipid; B Braun, Melsungen, Germany) for infusion of lipid-containing admixtures. In-line filters were arranged in each lumen of a central and peripheral venous catheter and were replaced after 24 hours (Intrapur Lipid/Intrapur Neonat Lipid) or 72 hours (ELD96LLCE/NOE96E) of regular use or in case of blockage. In both groups, administration sets for lipid-containing admixtures were changed every 24 hours, others every 72 hours as recommended by the Robert Koch Institute [17]. Both, nurses and physicians were extensively trained prior to the study to ensure correct and safe in-line filter handling. An open label study design characterized by visible in-line filters was necessary to ensure maximum safety in drug administration and to allow nurses to monitor the in-line filters for imminent blockage.

### Data collection

On admission, the paediatric index of mortality (PIM) II [18], demographic and clinical data were documented in databases for each patient. Blood chemical studies and hematologic tests were routinely performed at the time of admission, daily and when clinically required. Relevant clinical data were registered for each patient at least every hour. Several clinically endpoints including sepsis, SIRS, organ dysfunction etc. were prospectively documented in the database. The databases were thoroughly checked for consistency. Any queries were resolved and the final database entries were verified by investigators blinded for the allocation of the patients.

### Endpoints

The details of the primary and secondary endpoints (ClinicalTrials.gov number; NCT00209768) have been published previously [15]. Given the low mortality in the PICU, we chose reduction in the overall complication rate of major events as the primary endpoint. The study was designed using a chi-square test for equal proportions and with 80% power to detect a reduction of 10% in the complication rate for the filter group. Major events included the incidence of SIRS, sepsis, thrombosis, acute liver failure, ARDS, acute renal and circulatory failure. The occurrence of at least one of the preceding events during the PICU stay accounted for one complication per patient in the calculation of the overall complication rate. Complications prior to PICU stay and on admission were not taken into account.

In this investigation the occurrence of cardiovascular, respiratory, neurologic, hematologic, renal and hepatic dysfunction according to International Pediatric Sepsis Consensus Conference 2005 [16] (for detailed definition see Table 1) were analyzed in both, filter and control group during PICU stay. Organ dysfunction on admission or prior to PICU stay were not taken into account. 

**Table 1:** Criteria for organ dysfunction

	
**Cardiovascular**	Despite intravenous application of >40 ml/kg isotonic volume in 1 hour persisting:
	· Decrease in BP (hypotension) <5^th^ percentile for age or systolic BP < 2 SD below normal for age
	OR
	· Need for vasocative drugs to maintain BP in normal range (use of dopamine in dose >5 μg/kg/min or epinephrine, norepinephrine, or dobutamine at any dose)
	OR
	· Two of the following
	- Metabolic acidosis (base deficit >5 mmol/l)
	- Arterial lactate >2 times upper limit of normal
	- Oliguria: urine output <0.5 ml/kg/h
	- Prolonged capillary refill >5 sec.
	- Core to peripheral body temperature difference >3°C
**Respiratory**	· Oxygenation index <300 in absence of cyanotic heart disease or preexisting lung disease
	OR
	· PaCO_2_ >65 mmHg or increase of >20 mmHg over baseline
	OR
	· Proven need or FiO_2_ >0.5 in order to maintain saturation ≥92%
	OR
	· Need for nonelective mechanical ventilation (invasive or non-invasive)
**Neurologic**	· Glasgow Coma Scale (GCS) ≤11
	OR
	· Acute change in mental status with decrease in GCS ≥3 points from abnormal baseline
**Hematologic**	· Platelet count < 80.000/mm^3^ or decline of 50% in platelet count from highest value recorded over the past 3 days (for chronic hematology/oncology patients)
	OR
	International Normalized Ratio >2
**Renal**	Serum creatinine ≥ 2 times upper limit of normal for age or 2-fold increase in baseline creatinine
**Hepatic**	· Total bilirubin ≥ 4 mg/dL (not applicable for newborn)
	OR
	ALT 2 times upper limit of normal age

This table shows the diagnostic criteria for cardiovascular, respiratory, neurologic, hematologic, renal, and hepatic dysfunction according to the International pediatric sepsis consensus conference [16]. BP denotes blood pressure, GCS Glasgow Coma Scale, ALT alanine aminotransferase.

### Statistical analysis

Concisely, the trial was performed on an intention-to-treat basis to detect a reduction from 40 to 30% in the complication rate of major events for the filter group as the primary endpoint [15]. Major events included the incidence of SIRS, sepsis, thrombosis, acute liver failure, ARDS, acute renal and circulatory failure. Size and power of the study were not calculated for detection of a single reduction in organ dysfunction.

Baseline characteristics of the control and filter groups were compared using the t-test for equality of means. Differences between the two groups regarding the incidence rates and its 95% confidence interval (CI) (by the Wald method) were calculated for each organ dysfunction. Statistical analysis was performed using the Predictive Analysis Software for Windows (SPSS / PASW), version 18.

## Results

### Subjects

807 children, allocated to either control (n = 406) or filter group (n = 401), were included in the final analysis. As previously shown [15], there were no significant differences between both groups regarding baseline demographic characteristics, underlying disease categories, or Pediatric Index of Mortality II score (see Table 2). 

**Table 2:** Baseline characteristics of patients

**CHARACTERISTICS**	**CONTROL GROUP (n = 406)**	**FILTER GROUP (n = 401)**	**P VALUE**
**Age - years**	**5.58 ± 5.59**	**6.07 ± 6.01**	**0.23**
**Weight − kg**	**21.8 ± 20.1**	**23.0 ± 20.7**	**0.43**
**Paediatric Index of Mortality II (PIM II)**	**4.15 ± 8.76**	**3.42 ± 9.14**	**0.25**
**Sex − no.**			
Male	**230**	**234**	**0.72**
Female	**175**	**168**	
**DISEASE CATEGORY ON ADMISSION**			
**Cardiology**	**150**	**155**	**0.66**
*Cardiac bypass*	*101*	*102*	*0.87*
*non-bypass*	*49*	*53*	*0.67*
**Hematology/Oncology**	**24**	**21**	**0.76**
**Nephrology**	**18**	**26**	**0.21**
**Gastroenterolgy**	**37**	**37**	**1.00**
**Pulmonology**	**21**	**18**	**0.74**
**Pediatric surgery**	**59**	**48**	**0.30**
**Traumatolgy**	**34**	**43**	**0.28**
**Neurosurgery**	**26**	**22**	**0.66**
**Others**	**37**	**31**	**0.53**

This table displays the distribution of subjects between the control and filter groups by demographic characteristics, PIM II and disease categories on admission. None of the differences between the two groups were significant. Plus-minus values are means ± SD. PIM II denotes Pediatric Index of Mortality II. P values were calculated using the t-test for equality of means, Pearson’s Chi-Square test or Fisher’s exact test, as appropriate.

### Organ dysfunction

**Figure 1:**
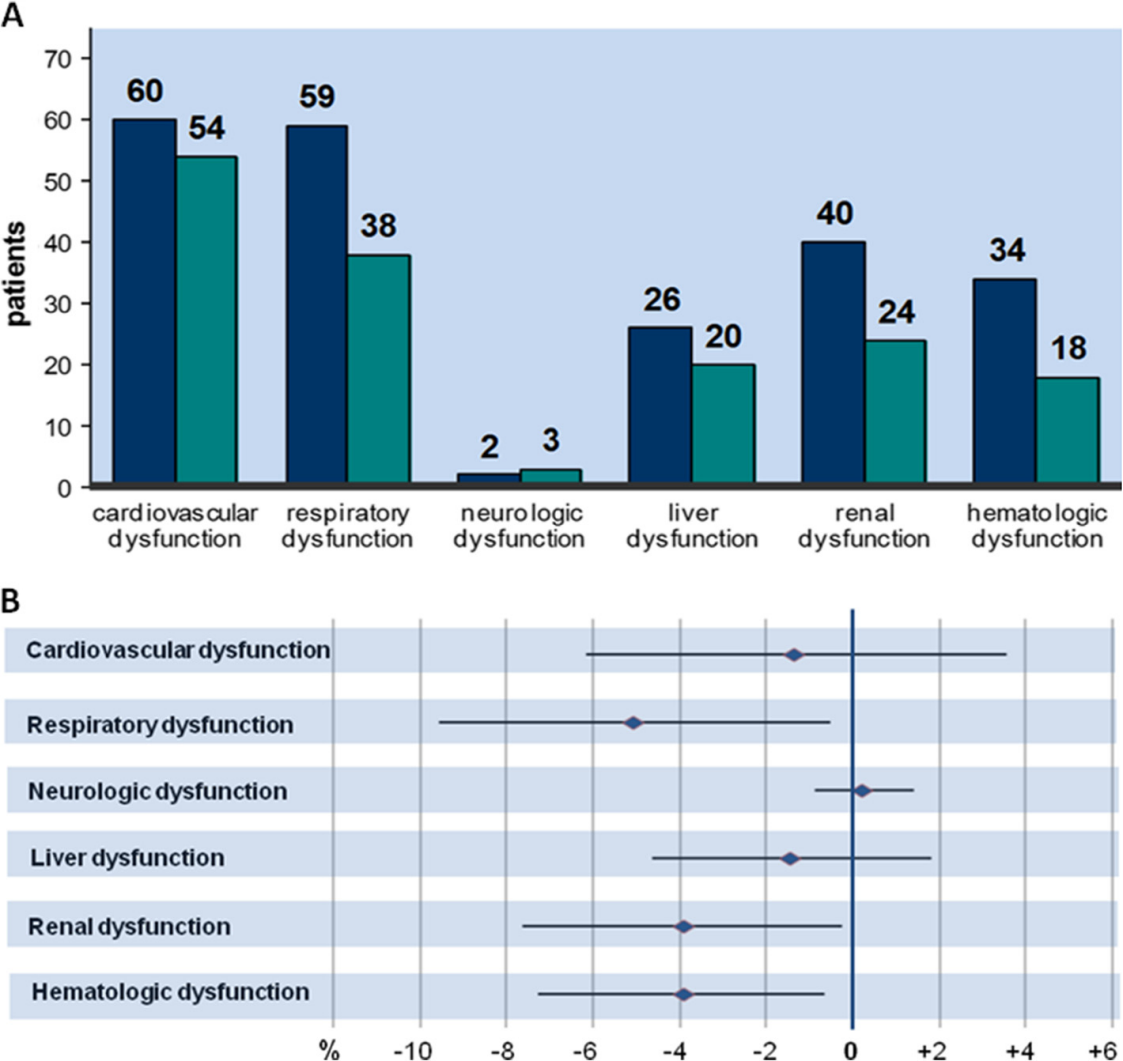
**Incidence of organ dysfunction in control (blue columns) and filter group (grey columns) (A) and corresponding differences in incidence rates with 95% confidence intervals (B).** Respiratory, renal and hematologic dysfunction were significantly reduced in the filter group (Panel **A**). Filled rhombi: differences in incidence rates; horizontal lines: 95% confidence intervals. (Panel **B**).

Patients receiving in-line filtration developed less frequently respiratory dysfunction (9.5% [n = 38] vs. 14.5% [n = 59]; difference in incidence rates = −5.06%; 95% CI, −9.52 to −0.59%; filter vs. control group), (Figure 1 and Table 3). Only 6.0% [n = 24] of the patients assigned to the filter group developed renal dysfunction, whereas 9.9% [n = 40] of the children of the control group suffered from renal dysfunction (difference in incidence rates = −3.87%; 95% CI, −7.58 to −0.15%; filter vs. control group). Moreover, in-line filtration reduced the incidence rate of hematologic dysfunction (4.5% [n = 18] vs. 8.4% [n = 34]; difference in incidence rates = −3.89%; 95% CI, −7.26 to −0.51%; filter vs. control group). No relevant differences were demonstrated for the incidence rates of cardiovascular (vs. 13.5% [n = 54] vs. 14.8% [n = 60]; difference in incidence rates = −1.31%; 95% CI, −6.12 to 3.49%), filter vs. control group), hepatic (5.0% [n = 20] vs. 6.4% [n = 26]; difference in incidence rates = −1.42%; 95% CI, −4.61 to 1.78%; filter vs. control group), and neurologic dysfunction (0.7% [n = 3] vs. 0.5% [n = 2]; difference in incidence rates = 0.26%; 95% CI, −0.83 to 1.34%; filter vs. control group) between both groups.

**Table 3:** Organ dysfunction

**CHARACTERISTICS**	**CONTROL GROUP (n = 406)**	**FILTER GROUP (n = 401)**	**Difference in incidence rates (%)**	**Confidence Interval (%) (Wald method)**
**Respiratory dysfunction**	**59**	**38**	**−5.06**	**−9.52 − −0.59**
**Renal dysfunction**	**40**	**24**	**−3.87**	**−7.58 − −0.15**
**Hematologic dysfunction**	**34**	**18**	**−3.89**	**−7.26 − −0.51**
**Cardiovascular dysfunction**	**60**	**54**	**−1.31**	**−6.12 − 3.49**
**Hepatic dysfunction**	**26**	**20**	**−1.42**	**−4.61 − 1.78**
**Neurologic dysfunction**	**2**	**3**	**−0.83**	**−0.83 − 1.34**

Table shows the incidence of the different organ dysfunctions and the resulting differences in the incidence rates and corresponding confidence intervals according to the Wald method.

## Discussion

As previously shown, in-line filtration was highly effective in reducing the overall complication rate of severe events (SIRS, sepsis, circulatory failure, acute respiratory distress syndrome (ARDS), thrombosis, acute renal and liver failure). Length of stay and duration of mechanical ventilation on the PICU were also significantly shortened if in-line filtration was applied [15]. In this additional investigation, the 95% CIs of the differences in the incidence rates of several organ dysfunction lay on either side below zero, indicating a statistically significant difference between both groups (Figure 1). The negative number in the difference of the incidence rates revealed a protective effect for the interventional group. Thus, in-line filtration may have a beneficial effect on the preservation of respiratory, renal and hematologic organ function in critically ill children. The incidence of other organ dysfunction was also lower in the filter group; however these differences did not reach statistical significance as the 95% CI included zero. The number of patients suffering from neurologic dysfunction was too small in both groups to allow any reliable statistical conclusion.

This current data support previous pathophysiological findings and confirm clinical and experimental data [5-8,10,12-15,19]. A reduced occurrence of respiratory dysfunction was evident for the filter group. These results are in accordance with our previous findings, which revealed a statistical trend towards a decrease in the incidence of ARDS for the filter group [15]. As a consequence, the duration of mechanical ventilation in patients receiving in-line filtration was shortened [15]. Since lung capillaries are the first anatomical filter for infused particles, the pulmonary vessels may be the primary cause for respiratory dysfunction as migration of particles to the lung induces mechanical embolisation of lung capillaries [7,19-22]. In adults suffering from ARDS, Walpot et al. demonstrated particulate-induced formation of occlusive microthrombi and generation of granulomas and foreign body giant cells on autopsy [19]. Particles may harm the pulmonary endothelium either directly or by activation of complement, platelets and/or neutrophils [7,19]. The infusion of particle-containing solutions could partially generate or at least aggravate ARDS and respiratory insufficiency.

However, the harmful effects of particles do not seem to be restricted to the lung as primary anatomical filter. Several authors have shown that inhaled [23,24] and intravenously injected [25] particles are translocated from the lung to the systemic circulation and various extrapulmonary organs, either as free particles or incorporated into macrophages. These particles can be detected in the blood, liver, kidney and the spleen. Also, particulate contaminants from drug preparations have been found, after intravenous injection traversing or bypassing the lung, in arterioles and capillaries of a striated muscle in a hamster skinfold chamber model [6,10]. After short ischemia-reperfusion injury, these particles damaged the microcirculation and induced a reduction of capillary density [6,10]. In critically ill patients with already compromised microcirculation, further impairment by particles may have additional deleterious effects on organ function [26]. Especially the renal function is highly dependent on vascular integrity. In critically ill patients, acute renal failure or dysfunction is a common problem in the course of sepsis, severe trauma, surgery, or shock, and is an independent risk factor for morbidity and mortality [27]. Renal dysfunction is mainly caused by insufficient tissue perfusion resulting in an ischemia–reperfusion injury with consecutive tubular injury or necrosis [27]. However, recently a new concept regarding the pathogenesis of acute renal failure has been evolved [28]. In sepsis-induced acute renal failure the renal blood flow is preserved and an increased renal vascular resistance as the pathogenetic factor leads to the development of acute renal failure or dysfunction [28]. This increased renal vascular resistance is mainly caused by an impairment of the microcirculation in the renal cortex and medulla. There, sepsis induces endothelial alterations with an adhesion of leukocytes and platelets, and a consecutive formation of microthromboses [28]. In the presence of such disturbed vascular integrity, particles may either trigger or augment alterations in the renal microcirculation. As shown in our study, patients provided with particle-retentive in-line filters had a lower incidence of renal dysfunction.

The incidence of hematological dysfunction (defined as thrombocytopenia below <80.000/mm^3^, decline in platelet count by more than 50% or international normalized ratio (INR) >2 [16]) was significantly decreased in the filter group. Clinically, thrombocytopenia alone or a drop in platelet count of >50% is associated with a raised mortality on intensive care unit [29]. The coagulation system — as in most critically ill patients [30] — seems to be more activated in patients of the control group compared to patients of the filter group. Particles obstructing small capillaries may increase platelet activation and consumption of clotting factors either directly or via interference with the endothelium with subsequent activation of complement, platelets and/or neutrophils [7,19]. This has been well illustrated for the lung as particles in capillaries as well as in the interstitium were surrounded by an accumulation of platelets and fibrin deposits [7,19]. In addition, also a systemic hypercoagulability, as proven for inhaled particles [31,32], can be hypothesized for intravenously injected particles after distribution to several organs.

Thrombogenic effects in the microcirculation induce and modulate an inflammatory activity [30]. Vice-versa, systemic inflammation results in activation of the coagulation [30]. Additionally infused particles may initiate and aggravate the cross-talk between the inflammatory and coagulation pathways and even prolong this vicious circle. In our study these synergistic effects of particles on the coagulation and inflammatory pathways become clinically obvious. Patients not being protected against particle infusion suffer from a higher incidence of SIRS [15] and haematological dysfunction. Additionally, as shown for septic patients the mutual activation of inflammation and coagulation results in an considerable increase of organ failure or dysfunction at multiple sites [33]. This resembles another potential pathophysiological explanation for the higher incidence of organ dysfunction in the control group.

In the control as well as in the filter group we used a standardized infusion regimen for all patients. In both groups medications were prepared according to the manufacturer’s instructions and parenteral nutrition and certain drugs were supplied by a centralized intravenous additive service to guarantee chemical stability and aseptic standards. The infusion therapy was further optimized by the use of a computer program to prevent formation of particles by precipitations and incompatibilities. It is remarkable, that although we used the best of practice for the infusion management in both groups, the additional use of in-line filtration resulted in a further positive effect for patients.

However, there is one inherent limitation of this study. Although, data for organ dysfunction was recorded prospectively, size and power of the study had not been calculated for the detection of a single reduction in organ dysfunction beforehand. Therefore, use of a contingency table and subsequent assessment of the significance by Pearson’s Chi-Square test or Fisher’s exact test with the computation of a P value would have been statistically incorrect. All data is therefore based on a descriptive statistical analysis. The presented results are related to the significant differences in the incidence rates and its 95% CI and have to be interpreted on the basis of the underlying descriptive statistic.

## Conclusion

In conclusion, our investigation demonstrated potential beneficial effects of in-line filtration on the preservation of various organ systems. The clinical data further support our hypothesis that infused particles act directly on the microcirculation of several organ systems such as the kidney and lung and induce a systemic hypercoagulability and inflammation. Further experimental and clinical investigations are necessary to clarify the exact impact as well as the underlying mechanisms of infused particles in order to identify their exact role in the development of complications in critically ill patients.

## Abbreviations

ARDS: Acute respiratory distress syndrome; CI: Confidence interval; INR: International normalized ratio; LOS: Length of stay; PICU: Pediatric intensive care unit; SIRS: Systemic inflammatory response syndrome; vs.: Versus.

## Competing interests

Funding was provided by a research grant from Hannover Medical School and partially by an unrestricted grant from Pall Corporation, Dreieich, Germany and B. Braun Corporation, Melsungen, Germany. Both companies supplied in-line filters during the study period. Dr. Jack, Dr. Sasse and Dr. Boehne report having been paid lecture and travel fees from Pall Corporation and B. Braun Corporation. No other potential conflict of interest relevant to this article was reported. The sponsors had no influence on the study design, patient enrolment, data collection, analyses, data interpretation or preparation of the manuscript. The trial was independently conducted by the investigators. Data were fully accessible to and interpreted by all authors, who attest to their accuracy and completeness. The authors had final responsibility for the decision to submit the manuscript.

## Authors’ contributions

TJ and MS designed the study. MB, TJ and MA were responsible for patient assessment and enrolment. MB, TJ, HK, KS, FS, HB and MS provided patient care and requested informed consent from the parents or legal guardians. MA, HK and FS and collected the clinical and laboratory data. MB and TJ checked data for consistency and performed the final validation. MB and TJ carried out the statistical analyses. All authors contributed to the interpretation of the results, the writing and critical review of the manuscript. All authors read and approved the final manuscript.

## Pre-publication history

The pre-publication history for this paper can be accessed here:

http://www.biomedcentral.com/1471-2431/13/21/prepub
